# An Atypical System for Studying Epithelial-Mesenchymal Transition in Hepatocellular Carcinoma

**DOI:** 10.1038/srep26282

**Published:** 2016-05-20

**Authors:** Dhiviya Vedagiri, Hiren Vasantrai Lashkari, Abubakar Siddiq Mangani, Jerald Mahesh Kumar, Jedy Jose, Avinash Raj Thatipalli, Krishnan Harinivas Harshan

**Affiliations:** 1 CSIR-Centre for Cellular and Molecular Biology, Hyderabad, 500007 India

## Abstract

Intrahepatic and extrahepatic metastases are frequently detected in hepatocellular carcinoma (HCC). Epithelial-mesenchymal transition (EMT) is believed to drive metastasis. There are not many well-established model systems to study EMT in HCC. Here we identified an atypical EMT while characterizing a population of mesenchymal cells in Huh7.5 hepatoma cell cultures. Cells with distinct morphology appeared during geneticin treatment of Huh7.5 cultures. Molecular characterization of geneticin resistant Huh7.5M cells confirmed EMT. Huh7.5M cells expressed cancer stem cell markers. p38MAPK and ERK1/2 were substantially activated in Huh7.5M cells. Their Inhibition elevated E-Cadherin expression with concerted suppression of Vimentin and anchorage independent growth in Huh7.5M cells. TGFβ could not induce EMT in Huh7.5 cultures, but enriched mesenchymal populations, similar to geneticin. Huh7.5M cells formed more aggressive solid tumors, primarily comprising cells with epithelial morphology, in nude mice. Canonical EMT-TFs did not participate in this atypical EMT, indicating that the established canonical EMT-TFs do not drive every EMT and there is a dire need to identify additional factors. The system that we characterized is a unique model to study EMT, MET and biphasic TGFβ signaling in HCC and offers considerable potential to facilitate more insightful studies on deeper questions in tumor metastasis.

Hepatocellular carcinoma (HCC) is the fifth most frequent malignant cancer worldwide and third most potent in cancer related mortality^1^. HCC has poor prognosis even after surgical removal of the tumor due to its successful vascular invasion and subsequent metastasis^2^,^3^. Being epithelial in nature, hepatocytes generate extensive extracellular matrix (ECM) forming a sheath like basement membrane (BM) and have strong cell-cell adhesion. They also have distinct basal and apical polarity. These properties are natural barriers for the cells to disseminate during metastasis.

Epithelial mesenchymal transition (EMT) has been identified as the process that facilitates carcinoma cells attain metastatic capabilities^4^,^5^. During EMT, epithelial cells lose their polarity, BM and cell-cell adhesion, and attain spindle like morphology providing greater flexibility for migration and subsequent invasion^6^,^7^. EMT in carcinomas has been demonstrated to generate cells with stem cell like properties^8^,^9^ and thus might be behind the generation of cancer stem cells (CSCs). Consistent with this theory, studies have identified circulating tumor cells (CTC) with EMT signatures^10^. Post-attachment to the foreign site, the mesenchymal cells are thought to convert back to its cancerous epithelial parental state through mesenchymal to epithelial transition (MET).

EMT is facilitated through transcriptional reprogramming by members of Snail, Zeb and Twist family of transcription factors (EMT-TFs)^7^,^11^. These transcriptional repressors target epithelial marker E-Cadherin^12^, which is a major adhesion molecule in epithelial cells. Loss of E-cadherin enables the release of carcinoma cells during metastasis. Among the other molecules suppressed during EMT are Zona Occludens-1 (ZO-1) and Claudin1. Loss of epithelial characteristics during EMT is concurrent with appearance of an array of mesenchymal markers such as Vimentin, N-Cadherin and β-Catenin.

TGFβ signaling pathway promotes EMT^13^,^14^,^15^. MAP Kinases (MAPKs) are key contributors as well^16^,^17^,^18^,^19^. TGFβ signals through its canonical SMAD pathway while non-SMAD pathways are also established^13^. Effect of TGFβ on cell fate is context dependent and unpredictable. Biphasic effects of TGFβ are well reported^13^,^20^. In primary epithelial cells, it promotes senescence while enhancing tumor aggression in carcinomas. There have been contrasting reports on the effect of TGFβ on HCC. Therapeutic use of TGFβ has been attempted with mixed outcomes^21^,^22^,^23^.

In the present study, we characterized a unique EMT in a sub-population of Huh7.5 hepatoma cell cultures. Through this report, we propose the existence of other EMT inducers in addition to the known EMT-TFs. We have identified an atypical EMT program that can be used in studies to address many pertaining questions in the field.

## Results

### Isolation of cells with distinct morphology from Huh7.5 cell culture

**Figure 1:**
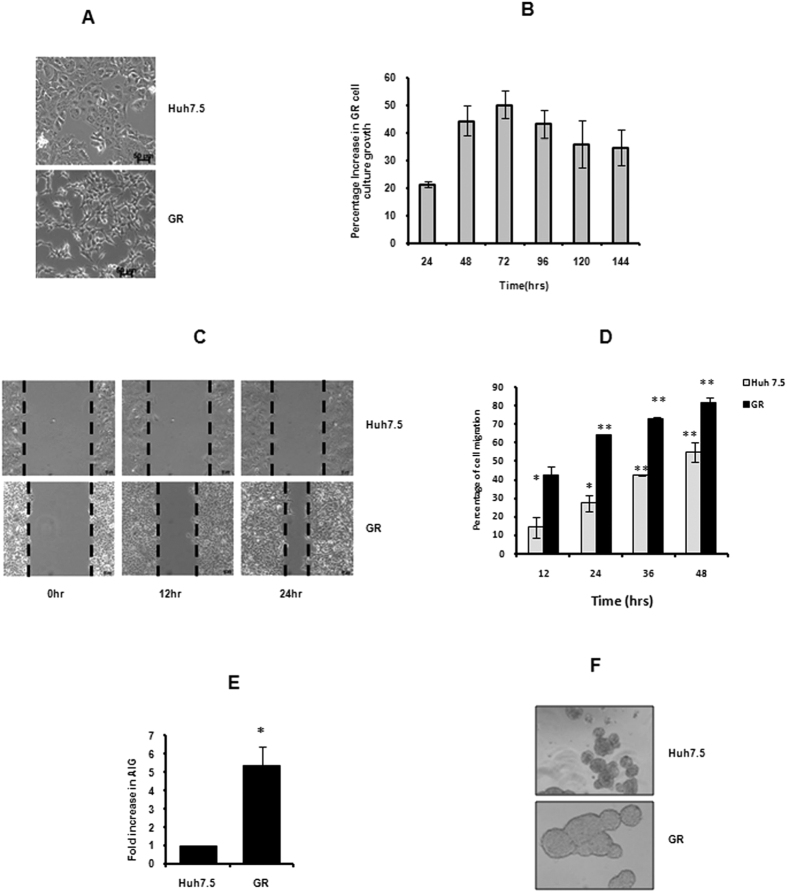
Characterization of GR cells. (**A**) Huh7.5 and GR cells under phase contrast microscope. (**B**) Proliferation of GR cells. Equal numbers of Huh7.5 and GR cells seeded on day 0 were cultured and cell counts were performed by trypan blue exclusion assay at specified time points. Percentage increases in GR cell count over that of Huh7.5 cells at specific intervals were plotted. (**C**) Images of wound healing assay. (**D**) Quantitative representation of wound healing. (**E**) AIG of cells grown in poly(HEMA) coated dishes assayed by MTT assay. Represented are the fold changes in MTT readouts. (**F**) Spheroid formation observed under phase contrast microscope. Statistical significance is shown at the top of the bars. ^*^p < 0.05; ^*^^*^p < 0.005. (**B**,**D**,**E**) are averages of at least three independent experiments.

We serendipitously came across geneticin resistant (GR) colonies in Huh7.5 hepatoma cell culture treated with geneticin as high as 2 mg/ml. While Huh7.5 cells are typically epithelial in appearance, the GR cells were significantly smaller with bright halo around, had characteristic spindle shape of fibroblastoid/mesenchymal cells and loose intercellular adhesion (Fig. 1A). They proliferated considerably faster than Huh7.5 cells (Fig. 1B). GR cells adhered loosely to cell culture substratum (laboratory observation) and migrated faster than Huh7.5 cells in wound heal assays (Fig. 1C,D). They displayed higher anchorage independent growth (AIG) (Fig. 1E) scores and augmented spheroid formation in polyHEMA coated dishes (Fig. 1F) than Huh7.5 cells. Interestingly, similar colonies could not be generated by other commonly used antibiotics such as blasticidin, zeocin and puromycin.

### A unique EMT generates GR cells

**Figure 2:**
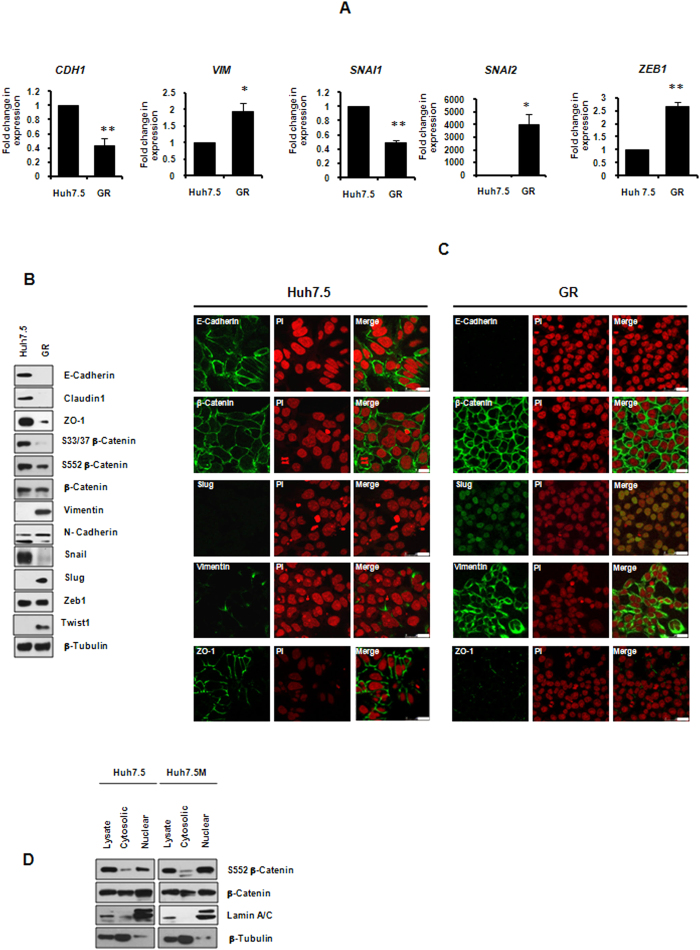
GR cells are generated through an atypical EMT. (**A**) qRT-PCR analysis of EMT markers in Huh7.5 and GR. (**B**) Molecular characterization of EMT markers in Huh7.5 and GR cells by immunobloting. (**C**) Localization of EMT makers in Huh7.5 and GR cells by confocal immunofluorescence. (**D**) Nuclear localization of β-Catenin in Huh7.5 and Huh7.5M cells. Total cell lysates, cytoplasmic and nuclear fractions were analyzed for the presence of β-Catenin by immunobloting. All graphical images are averages of at least three independent sets of results. ^*^p < 0.05; ^*^^*^p < 0.005.

We speculated that EMT is behind geneticin resistance as the process is associated with drug resistance in tumors. mRNA quantitation by qRT-PCR indicated transcriptional reprogramming (Fig. 2A). Huh7.5 cells expressed higher levels of *CDH1* and *SNAI1* while *VIM, SNAI2* and *ZEB1* were expressed excessively in GR cells. Immunobloting confirmed the results of qRT-PCR. E-cadherin, Claudin1 and ZO-1 were suppressed in GR cells as compared to Huh7.5 (Fig. 2B). Again, Snail was predominantly detected in Huh7.5 cells than in GR cells. Vimentin, Twist1 and Slug were expressed robustly in GR cells. In spite of its abundance at mRNA levels in GR cells, Zeb1 expression varied considerably and did not show any cellular allegiance. Largely, N-Cadherin and β-Catenin expressions did not reveal consistent demonstrable change.

Next, localization of markers was analyzed by confocal immunofluorescence microscopy. Absence of E-Cadherin and ZO-1 was evident in GR cells (Fig. 2C). β-Catenin was retained on membranes in both the cell types. While Slug remained localized to nuclei, Vimentin was detected throughout GR cells. In combination with immunobloting results, these results demonstrate that GR cells were indeed generated because of EMT in Huh7.5. From here on, GR cells are referred to as Huh7.5M (Huh7.5-mesenchymal). We consider E-Cadherin and Claudin1 as faithful epithelial, and Slug and Vimentin as mesenchymal markers in this system.

β-Catenin is a well-established modulator in wnt signal pathway, which is a major regulator of EMT. Its activity is regulated by its phosphorylation at multiple residues. S33/37 phosphorylation by GSK-3β renders β-Catenin to degradation while S552 phosphorylation promotes it nuclear translocation, facilitating its transcriptional activity. S33/37 phosphorylation was lower in GR cells as compared with Huh7.5 cells, suggesting that β-Catenin is more stable in them (Fig. 2B). However, S552 phosphorylation was also inhibited in GR cells. Thus, increased stability gained by lower S33/37 phosphorylation was probably not translated into its nuclear localization. Next, its nuclear translocation was studied by nuclear fractionation followed by immunobloting. As demonstrated in Fig. 2D, both cells displayed robust localization of β-Catenin in nuclei. These results are in agreement with immunofluorescence data (Fig. 2C) and they collectively suggest that β-Catenin and possibly wnt signaling is not playing a major role in this system.

### Huh7.5M cells express cancer stem cell markers

**Figure 3:**
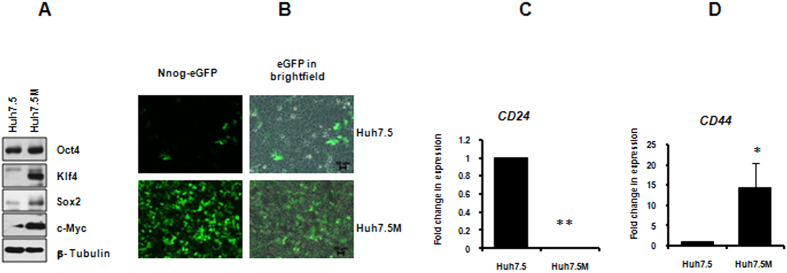
Huh7.5M cells have CSC properties. (**A)** Analysis of expression of pluripotency markers by immunobloting. (**B**) Expression of eGFP from *NANOG*-promoter reporter vector. Huh7.5 and Huh7.5M cells transfected with PL-SIN-Nanog-EGFP were imaged using a fluorescence microscope. (**C**) qRT-PCR analysis of *CD24* and (**D**) that of *CD44* in Huh7.5 and Huh7.5M cells. Represented are averages from a minimum of three independent experiments.

CSCs are believed to be generated by EMT. Huh7.5M cells expressed elevated levels of Yamanaka factors Sox-2, Klf4 and c-Myc (SKM) as compared with Huh7.5 cells (Fig. 3A). *NANOG* promoter showed remarkably higher activity in Huh7.5M cells than Huh7.5 cells, as demonstrated by increased eGFP expression driven by *NANOG* promoter (Fig. 3B). *CD44*^high^/*CD24*^low^ phenotype is associated with many CSCs. qRT-PCR revealed *CD44*^high^/*CD24*^low^ phenotype in Huh7.5M cells as compared to the *CD44*^low^/*CD24*^high^ phenotype in Huh7.5 (Fig. 3C,D). Thus, Huh7.5M cells are CSCs generated through EMT.

### TGFβ fails to induce EMT, but enriches rare CSC population in Huh7.5 culture through biphasic signaling

**Figure 4:**
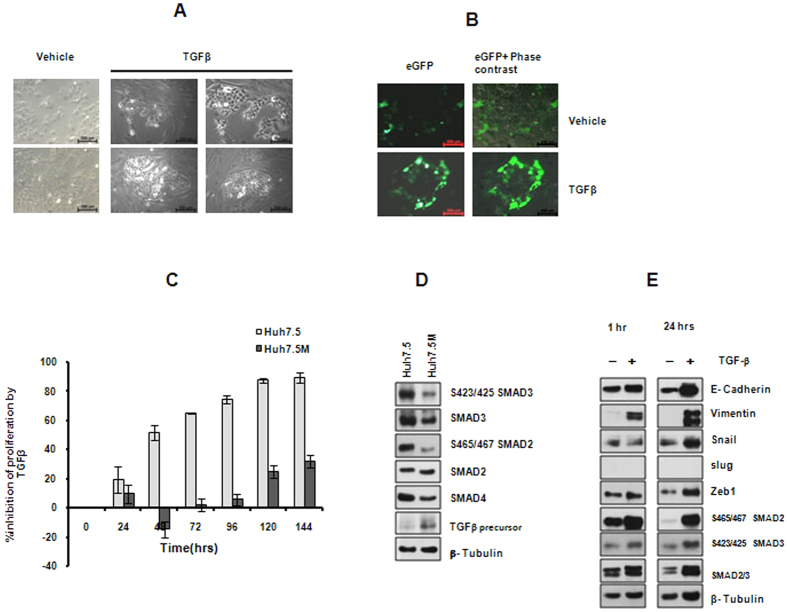
Effect of TGFβ treatment on Huh7.5 cells. (**A**) Huh7.5 cells were treated with 2 ng/ml of TGFβ or with vehicle for seven days. Colonies appeared upon treatment were imaged. (**B**) Huh7.5 cells were transfected with PL-SIN-Nanog-EGFP plasmid followed by TGFβ or vehicle treatment at 24 hours post-transfection. Images were captured using a fluorescent microscope. (**C**) Effect of TGFβ on proliferation of Huh7.5 and Huh7.5M cells. Percentage of growth inhibition by TGFβ over the vehicle control is represented. (**D**) Analysis of SMAD2-3 signaling in Huh7.5 and Huh7.5M cells. (**E**) Effect of TGFβ treatment on SMAD2–3 signaling in Huh7.5 cells analyzed by immunobloting. Cells were treated with 2 ng/ml of TGFβ for 1 hr or for 24 hrs before harvesting them for analysis.

Since TGFβ is a well-known inducer of EMT, we asked if it causes EMT in Huh7.5 cells. Upon treatment with 2 ng/ml of TGFβ for a week, majority of Huh7.5 cells underwent cell death while a few colonies, encompassing cells that were morphologically similar to Huh7.5M cells, emerged (Fig. 4A and Fig. S1). The emerging colonies appeared characteristically oval shaped, surrounded by elongated cells that subsequently underwent cell death. Thus, TGFβ enriches a unique population of de-differentiated cells that transited into mesenchymal format. Confined expression of eGFP from *NANOG* reporter vector to the cells in TGFβ enriched colonies further confirmed the stemness of TGFβ enriched cells (Fig. 4B). As expected, Huh7.5M cells exhibited remarkable resistance to TGFβ induced apoptosis (Fig. 4C). Thus, TGFβ appears to be enriching the rare mesenchymal cells in the population instead of inducing widespread EMT in Huh7.5 cells. Smad 2 and 3 phosphorylations were considerably suppressed in Huh7.5M cells (Fig. 4D), in spite of higher TGFβ precursor expression (Fig. 4D). TGFβ treatment of Huh7.5 cells for 24 hrs resulted in concurrent activation of E-Cadherin, Snail and Vimentin (Fig. 4E). Thus, TGFβ does not activate canonical EMT program in Huh7.5 cells.

### p38MAPK and ERK1/2 independently regulate EMT in Huh7.5M cells

**Figure 5:**
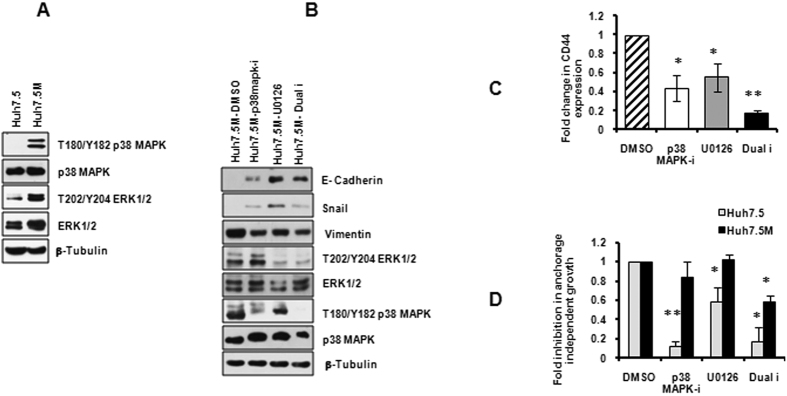
p38MAPK and ERK1/2 regulate EMT in Huh7.5-Huh7.5M system. (**A**) Phosphorylation of p38MAPK and ERK1/2 in Huh7.5 and Huh7.5M cells were analyzed by immunobloting. (**B**) Effects of p38MAPK (50 μM) and ERK1/2 (100 μM) inhibition on EMT markers in Huh7.5M cells studied by immunobloting. Huh7.5M cells were treated with the inhibitors for 1 hr before analysis. (**C**) qRT-PCR analysis of *CD44* expression in Huh7.5M cells upon inhibition of p38MAPK and ERK1/2 for 1 hr. (**D**) Inhibition of AIG by the inhibitors studied by MTT assay. Both (**C,D**) depict average of results from three independent experiments. ^*^p < 0.05; ^*^^*^p < 0.005

p38MAPK and ERK1/2 are frequently activated in many high-grade tumors. p38MAPK is critical for TGFβ mediated EMT^24^. ERK 2 was reported to induce EMT through transcriptional activation of Zeb1/2 in breast cancer cells^25^. Inhibiting ERK1/2 signaling in lung cancer cells prevented EMT^18^. Both p38MAPK and ERK1/2 phosphorylations were found to be substantially activated in Huh7.5M cells over Huh7.5 cells (Fig. 5A).

To investigate their possible regulation of EMT, Huh7.5M cells were incubated independently with predetermined concentrations of 50 μM of p38MAPK inhibitor VIII or 100 μM U0126 for 1 hr and in combination. EMT markers responded remarkably similar upon p38MAPK and ERK1/2 inhibitions. Importantly, inhibition of both molecules enhanced E-Cadherin expression while suppressing Vimentin (Fig. 5B). Dual treatment failed to augment the effects. In addition, *CD44* was substantially suppressed by both inhibitors (Fig. 5C). Further, regulation of AIG by p38MAPK and ERK1/2 pathways was investigated. Huh7.5 and Huh7.5M cultures in poly(HEMA) coated cells were treated with the inhibitors at concentrations described above. Inhibitors were supplemented every 24 hrs and AIG was analyzed by MTT assay at the end of 72 hrs of treatment. Individually, both inhibitors remarkably inhibited AIG in Huh7.5 cell, but were inefficient in Huh7.5M cells (Fig. 5D). However, in combination, the drugs inhibited AIG considerably in Huh7.5M cells as well. These results suggest that p38MAPK and ERK1/2 contribute independently as well as synergistically to regulate EMT. Our results indicate that p38MAPK and ERK1/2 pathways regulate EMT in Huh7.5 cells and their activation could potentially play major roles in EMT in hepatocellular carcinoma.

### Canonical EMT-TFs do not cause EMT in Huh7.5 cells

**Figure 6:**
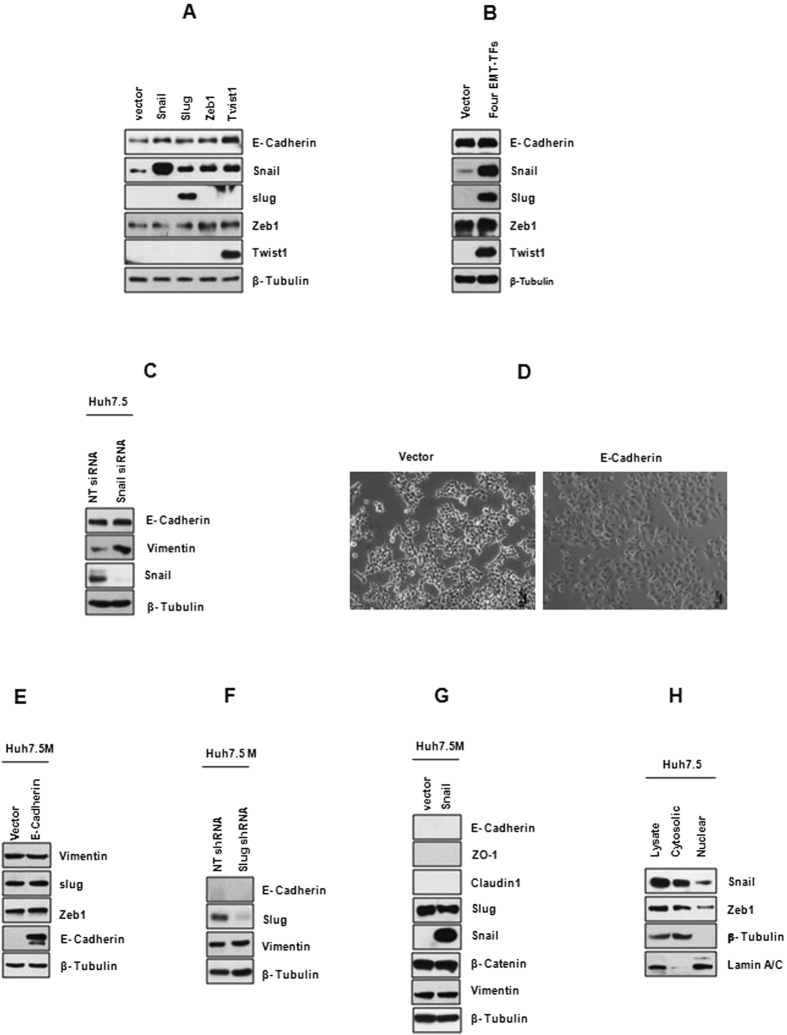
EMT-TFs do not regulate EMT in Huh7.5 cells. (**A**) Effect of ectopic expression of EMT-TFs on EMT markers in Huh7.5 cells was analyzed by immunobloting. Huh7.5 cells were transfected with empty vector pcDNA4/TO or that expressing one of the EMT-TFs. 48 hrs later, cells were harvested and analyzed. (**B**) Similar to (**A**), but the four EMT-TFs were co-expressed in Huh7.5 cells and their effect on EMT was analyzed by immunobloting. (**C**) Effect of Snail depletion on E-Cadherin expression in Huh7.5 cells. Huh7.5 cells were transfected with siRNA pool targeting Snail or with non-targeting pool (NT) and 72 hrs later, cells were harvested for analysis by immunobloting. (**D**) Effects of ectopic expression of E-Cadherin in Huh7.5M cells studied under microscope and (**E**) by immunobloting. As described in (**A**), Huh7.5M cells were transfected with E-Cadherin expression plasmid or with empty vector and 48 hrs post-transfection, cells were analyzed. (**F**) Effects of Slug depletion in Huh7.5M cells on EMT. shRNA pools targeting Slug or NT pool were transfected into Huh7.5M cells. 72 hrs later, cells were harvested for analysis. (**G**) Effect of Snail expression in Huh7.5M cells. Huh7.5M cells were transfected with Snail over-expressing plasmid or with empty plasmid. 48 hrs later, cells were analyzed by immunobloting. (**H**) Nuclear translocation of EMT-TFs in Huh7.5. Nuclear and cytosolic fractions from cultured Huh7.5 cells were immunobloted to detect the presence of EMT-TFs inside nuclei.

In Huh7.5 cells, Snail and Zeb1 are basally co-expressed with E-Cadherin. They are also concurrently over-expressed upon TGFβ treatment. In addition, inhibition of p38MAPK and ERK1/2 activated simultaneous expression of E-Cadherin and Snail. As these patterns are contrary to the current understanding of E-Cadherin suppression by EMT-TFs, we asked if the canonical EMT-TFs are functionally defective in Huh7.5 cells. Forced expression of individual EMT-TFs not only did not suppress E-Cadherin, but also marginally elevated it (Fig. 6A). Huh7.5 cells stably expressing any of these TFs except Zeb1 behaved similarly to the control cells, as in the case of transient expression studies (data not shown). Zeb1 stable expression could not be achieved. Co-expression of Snail, Zeb1, Slug and Twist1 also failed to suppress E-Cadherin (Fig. 6B). Vimentin levels also remained unchanged during these experiments (data not shown). Depletion of Snail in Huh7.5 cells caused little change in E-Cadherin expression (Fig. 6C). These results reassert the authenticity of Snail and E-Cadherin co-existence in Huh7.5 cells while ruling out functional mutations in the endogenous EMT-TFs. E-Cadherin, when expressed in Huh7.5M cells, brought about partial epithelial morphology with no effect on Vimentin (Fig. 6D,E). Additionally, Slug depletion in Huh7.5M cells also failed to alter molecular characteristics (Fig. 6F). Further, ectopic expression of Snail failed to activate epithelial features in Huh7.5M (Fig. 6G). Collectively, these results imply the non-involvement of the EMT-TFs in EMT of Huh7.5M.

Next, nuclear localization of the EMT-TFs was examined to study if mislocalization could be behind their inefficacy in EMT. Both Snail and Zeb1 were detected in nuclear fraction of Huh7.5 cells (Fig. 6H), while Slug was shown earlier in the nuclei of Huh7.5M cells (Fig. 2C). Therefore, we conclude that localization defects of the EMT-TFs are not responsible for their inability to induce EMT.

### Huh7.5M cells form aggressive tumors

**Figure 7:**
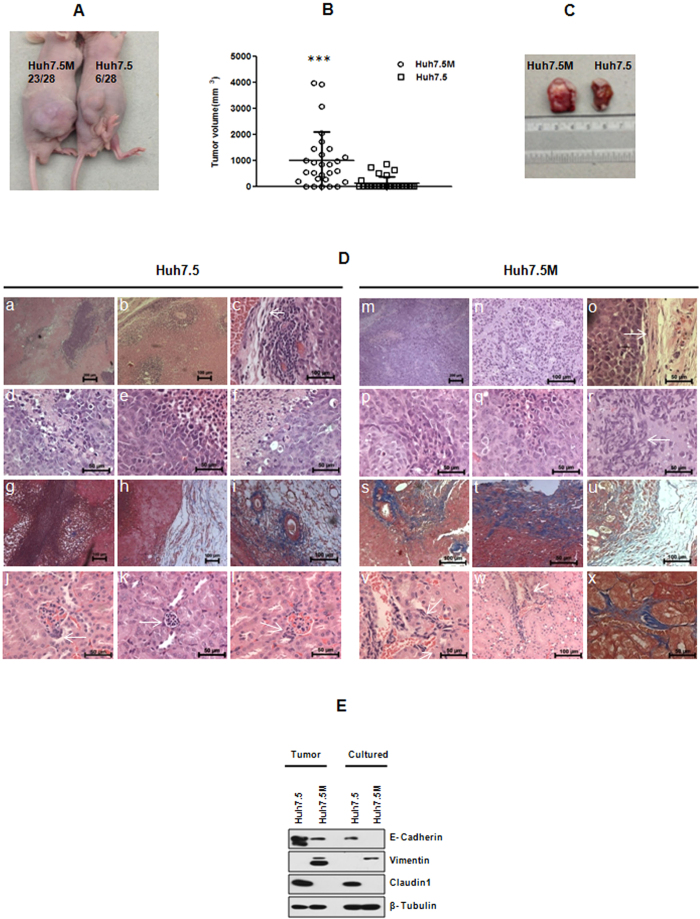
Huh7.5M cells form aggressive tumors. **(A**) Representative images of Nude mice with tumors generated from Huh7.5 and Huh7.5M cells. The numbers of animals that developed tumor and the total number of animals injected are given. (**B**) Analysis of tumor volume in nude mice. Each spot represents individual mouse. ^***^p < 0.0005. (**C**) Representative image of tumors. (**D**) Histopathological analysis of tumor tissues and kidney from injected animals. Samples were processed and stained with either HE or MT. Images a–f, m–r, j–l, v and w represent HE staining while g–i, s–u and x represent MT staining. j–l and v–x represent kidneys from animals injected with Huh7.5 and Huh7.5M respectively. (**E**) Comparison of EMT marker expression in tumors and tumor-derived cells by immunobloting.

In mouse xenograft studies, Huh7.5M cells generated substantially larger tumors at remarkably higher frequencies than Huh7.5 cells did (Fig. 7A–C). While majority of mice injected with Huh7.5M cells developed primary tumors, only a small set of animals developed tumors by Huh7.5 cells (Fig. 7A,B). Huh7.5-tumors contained extensive necrotic regions than Huh7.5M-tumors (compare Fig. 7D a,b and g with m,n). Interestingly, Huh7.5M-tumors largely contained cells with epithelial morphology (Fig. 7D p,q), as in the case of Huh7.5-tumors (Fig. 7D d,e). Invasive fronts (Fig. 7D o,t,u) and certain highly disordered regions (Fig. 7D n,r) in Huh7.5M tumors contained mesenchymal like cells. Masson’s Trichrome staining confirmed mesenchymal nature of cells in such regions (Fig. 7D s–u). Huh7.5-tumors also contained mesenchymal cells in invasive fronts (Fig. 7D c,h,i). Huh7.5M-tumors extensively metastasised to kidneys (Fig. 7D v–x) while this was also displayed at considerably lower frequency by Huh7.5 cells (Fig. 7D j–l). Invasion was detected in kidneys of Huh7.5M-tumor mice more frequently than in their Huh7.5 control groups. Migration into interstitium was observed in kidneys of Huh7.5M-tumor mice (Fig. 7D v–x). Huh7.5M-tumors metastasised to liver in rare instances (Fig. S2). Thus, Huh7.5M cells formed more aggressive tumors than Huh7.5 cells and contained higher preponderance of migration to kidneys.

Huh7.5M-tumors expressed moderate levels of E-Cadherin, while retaining Vimentin expression (Fig. 7E). Huh7.5-tumors expressed robust levels of E-Cadherin, but no Vimentin. Notwithstanding the expression of E-Cadherin, Claudin1 remained suppressed in Huh7.5M-tumors. Expression of these markers in tumor-derived cells after culturing followed their respective pattern as seen in cultured cells. Thus, Huh7.5M cells underwent partial MET *in vivo*.

## Discussion

EMT in cancer is a robust, dynamic process that prepares the cells to respond to various signals to facilitate its survival. The cells gain tremendous plasticity through this process resulting in a highly heterogeneous population of cancer cells in solid tumors. Understanding EMT to the fullest is a great challenge as the magnitude of EMT varies from partial to complete. Through this report, we are introducing a valuable, albeit atypical, system for thorough study of EMT-MET in HCC to the community. Currently, there are not many such systems available to the researchers to study metastasis in HCC. Indeed, a few non-metastatic and some metastatic cell lines are available for studies. However, a major deficiency is the lack of availability of the parental cell line of the mesenchymal lineage. They are either available as metastatic or non-metastatic cell lines that were originally generated from tumor samples. Then there are epithelial systems where EMT can be induced through TGFβ or by EMT-TFs. The easily culturable epithelial Huh7.5 cells and their mesenchymal Huh7.5M derivatives would constitute a valuable pair of tools to study either sides of EMT. This pair would greatly assist studies to understand fundamental changes that epithelial carcinoma cells undergo during its acquisition of metastatic capabilities. In addition, this system has a set of novel features that would make the studies more interesting. They are (i) the absence of involvement of canonical EMT-TFs (ii) biphasic signaling induced by TGFβ, resulting in enrichment of the unique CSC population and (iii) a partial MET that is observed only in primary tumor *in vivo*.

A far-reaching message from our study is that regulators other than the canonical EMT-TFs do exist. The absolute futility of canonical EMT-TFs in EMT is very evident in this system. We demonstrate here that co-existence of EMT-TFs with E-Cadherin is possible in epithelial background. EMT-TFs not only could not induce EMT in Huh7.5 cells, but concurrent activation of Snail and E-Cadherin expressions was also observed in Huh7.5 cells during TGFβ treatment, and during inhibition of p38MAPK and ERK1/2 in Huh7.5M cells. Thus, transcriptional regulatory networks in Huh7.5 cells seem to be distinctly different from that of the other reported EMT systems. The plasticity in enabling diverse mechanisms of EMT highlights its adaptability and contributes to the heterogeneity of cancer cell populations.

Why are EMT-TFs incompetent to regulate EMT in Huh7.5 cells? Are they not targeted to their canonical promoters? Are accessory regulatory proteins absent? It is possible that more than one factor is required to initiate EMT. However, co-expression of the four EMT-TFs still failed to induce EMT in our experiments. Are they performing some functions at all? Are their functions intercepted by other regulatory pathways? Canonical EMT-TFs have distinct requirement of accessory proteins and the odds of all of them totally missing is low. Even though E-Cadherin and Snail are concurrently expressed in many conditions in this report, Snail does not regulate E-Cadherin expression, as its over-expression failed to induce E-Cadherin expression in Huh7.5M cells. Epigenetic changes in E-Cadherin promoter is well-recognized^26^,^27^ and epigenetic plasticity is a major contributor to heterogeneity among tumor cells. Histone deacetylases (HDACs) have been demonstrated to regulate EMT in tandem with EMT-TFs^28^,^29^. We identified that HDAC 1, 6 and 8 are over-expressed in Huh7.5M cells against Huh7.5 cells (Fig. S6B). However, SAHA, a pan-HDAC inhibitor caused suppression of E-Cadherin and activation of Vimentin in Huh7.5M cells, indicating the necessity of careful analysis (Fig. S6C). Further studies are required to identify the roles of these molecules in EMT in Huh7.5M cells.

Through convincing evidences, we argue for the existence of other “key players” or “non-canonical mechanisms” in EMT. According to the current understanding, EMT is induced by TGFβ and wnt signal pathways. Both pathways crosstalk and converge at one of the canonical EMT-TFs through which EMT is executed. Wnt signal pathway is a good candidate to examine, but β-Catenin phosphorylation and localization studies did not provide encouraging leads. mTORC1 was demonstrated to maintain “epithelialness” in breast cancer cells and its suppression induced EMT in breast cancer cell lines independent of TGFβ. However, this mechanism required the activation of EMT-TFs Zeb1 and Zeb2^30^. Currently, we are unsure of the involvement of mTORC1 in Huh7.5M EMT. However, we also have strong evidence that the mechanism is under the regulation of p38MAPK and ERK1/2.

Prrx1 induces EMT and members of Ets family were shown to regulate EMT^31^,^32^,^33^,^34^. However, these inducers either act in tandem with or are regulated by one of the canonical EMT-TFs or TGF-β. Both *Prrx1* and *Ets1* were expressed more robustly in Huh7.5M cells than in Huh7.5 cells (Fig. S6A). To the best of our knowledge, this is the first report categorically demonstrating the non-involvement of canonical EMT-TFs in EMT. Unarguably, EMT circuitry in Huh7.5 cells is distinctly different from the canonical ones identified so far. It would be critical to identify the possible mechanism driving EMT in Huh7.5M cells. At present, what is evident is that this mechanism would be independent of the known EMT-TFs and TGFβ. A study that sought to identify core EMT gene signatures revealed a common EMT signature among mammary epithelial cells upon ectopic expression of Snail, Twist, Goosecoid (GSC) or TGFβ treatment^35^. Significant divergences were also identified between these individual treatments, suggesting the existence of unique and shared processes during EMTs induced by various factors. Thus, EMT inducers such as GSC, Ladybird homeobox 1, LIM homeobox genes and left-right determination factor (*LEFTY1*) would need to be studied in detail. Similar molecules have been reported in HCC as well^36^.

Parallels have been drawn between EMT in embryo development and cancer progression^9^,^37^, facilitating exchange of ideas between the two major fields of biological research. Genome-level expression or knock-down studies would be necessary to identify the factors that drive EMT in Huh7.5M cells. This should be possible with efficient CRISPR tools and suitable reporter markers such as Vimentin and E-Cadherin. A whole transcriptome profiling of Huh7.5 and Huh7.5M cells, coupled with genome level expression of knock-down studies would shed more light in deciphering the mechanism by identifying the key factors behind EMT in Huh7.5M cells. Possibilities of identifying either new EMT-TFs or co-factors exist. Therefore, this study expands the scope of hunting for unreported EMT inducers.

Biphasic effect of TGF-β was spectacularly displayed in our system. This unique observation is distinct from other TGFβ responsive metastatic tumor systems where the whole population undergoes EMT upon TGFβ treatment. Differential effects of TGFβ on HCC are documented^38^. Authentic evidences point to the existence of different subsets of tumor initiating cells giving rise to distinct subtypes of cancer. Our study demonstrates that existence of subset of cells similar to Huh7.5M in liver tumors would generate more aggressive and drug resistant tumors upon TGFβ treatment. Understanding the fundamental differences in TGF-β signaling between these populations is critical before TGFβ can be used as a therapeutic agent or a target. The system characterized by us would complement investigations in this direction. Geneticin and TGFβ enriched mesenchymal population with remarkable similarity. Remarkably, geneticin treatment of Huh7.5 cells induced TGFβ signaling (Fig. S4) and thus the antibiotic might functionally mimic TGFβ and promote enrichment of mesenchymal cells. Importantly, not every single treatment with either molecule generated Huh7.5M like cells, suggesting that the cells that responded positively were unique and generated in culture spontaneously through genetic or epigenetic modifications. It is unlikely that either of the molecules promoted EMT in such populations, but simply eliminated the rest of the population, allowing the CSCs to populate. Thus, generation of CSCs in this system follows a stochastic model.

Transient acquisition of epithelial phenotype displayed by Huh7.5M-tumor cells is significant. Re-expression of E-Cadherin in secondary tumors that originated from primary tumors lacking its expression has been reported^39^. *In vitro*, Huh7.5M cells have been uncompromising against MET, except during E-Cadherin expression. However, they underwent a transient MET in mice. Notwithstanding their epithelial morphology and E-Cadherin expression, Huh7.5M-tumor derived cells readily converted to mesenchymal phenotype when cultured *in vitro* (Fig. S3). Thus, at least a subgroup of Huh7.5M-tumor cells is in a more fluidic hybrid “transit” state retaining both epithelial and mesenchymal properties and is well poised to switch between the two formats. Existence of such hybrid state has recently been reported^40^ and is a proposed mechanism of survival of CTC clusters^10^. Clearly, the extrinsic factors in the *in vivo* niche that confers them epithelial features are critical. The mechanism by which CTCs undergo MET to form secondary tumors is predictably the same as we observe in our studies. Understanding the details of this “transit” state would be crucial in unraveling the secrets of EMT and MET.

In spite of their lower aggression in primary tumorigenesis, Huh7.5-tumors also metastasized to kidney, albeit at lower efficiency, suggesting that some cells in the population transited to mesenchymal phenotype. Such cells were seen in the invasive fronts of these tumors. Notwithstanding their similarity in morphology with Huh7.5-tumor cells, Huh7.5M-tumor cells were distinctly different as they produced aggressive tumors at higher frequency. Tumor initiating mechanisms followed by the two cell types in mice could be clearly distinct from each other. This could explain the phenomenally lower tumor initiation capacity of Huh7.5 cells. Huh7.5 cells continued their epithelial lineage while Huh7.5M cells transiently acquired epithelial properties.

Inhibition of ERK1/2 keeps hepatocytes in differentiated state^41^. ERK1/2 has also been reported to promote HCC^42^. However, its contribution to HCC metastasis is not clear. Notwithstanding E-Cadherin expression in Huh7.5M cells upon individual inhibition, p38MAPK and ERK1/2 inhibitions could not inhibit AIG alone, indicating that E-Cadherin expression and AIG are on separate circuits of regulation. Clearly, the two molecules target unique as well as common downstream molecules. It is evident from our results that the two kinases hold substantial control over EMT and their inhibition activated epithelial program in the mesenchymal format.

Divergent reports exist in literature on the roles of EMT-TFs from mouse Xenograft studies and clinical studies in HCC^43^,^44^,^45^,^46^. Some reports show a strong correlation between Snail and HCC and with poor prognosis^44^,^46^. Majority of these analyses with clinical samples have used E-Cadherin as the sole epithelial marker for establishing the identity of cells. Even though E-Cadherin loss indicates onset of EMT, it cannot measure the extent of EMT and thus cannot be directly correlated to tumor aggression. An early study that screened expression of E-Cadherin in a number of HCC cell lines only detected it in HepG2^47^. However, the study did not include Huh7 or its lineages. We also detected similar inverse correlation between Snail and E-Cadherin in HepG2 while Huh7, the parental cell line of Huh7.5, displayed direct correlation as in Huh7.5 (Fig. S5). Additionally, TGFβ induced the “transit” state in Huh7.5 cells by augmenting the expressions of E-Cadherin and Vimentin simultaneously. TGFβ also induced the expression of both Snail and Zeb1 in Huh7.5 cells, once again confirming the inability of these factors on EMT. These observations highlight the diversity and heterogeneity in HCC tumors, and advocate for more extensive coordinated studies employing clinical samples, mouse xenografts and cell culture. Careful cataloguing of markers is prerequisite for tumor grading.

Huh7.5 cells are descendents of non-metastatic Huh7 cells^48^. Huh7.5 cells were generated through extensive treatment of Huh7 cells with interferons. In the process, the cells might have accumulated mutations that might promote EMT. However, no substantial change was detected in the expression of markers between Huh7 and Huh7.5 cells (Fig. S5), suggesting that Huh7.5 cells retain basic epithelial characteristics of Huh7 cells. TGF-β and geneticin failed to induce EMT in Huh7 cultures in our hands (laboratory observations). Cells accumulate genetic and epigenetic alterations in cultures faster than in organisms. Understanding these changes with the help of such cells could be vital in unraveling the mechanisms behind biological processes. Our results are major advancement in the field and lessons learned from this system could be extremely useful in other EMT systems as well. Here we present an atypical system of EMT in metastasis that would be appealing to HCC researchers and to those in cancer biology in general by providing avenues to addressing questions on (i) unknown mechanisms of EMT, (ii) biphasic signaling by TGFβ in HCC and (iii) switching between EMT and MET *in vivo*.

## Methods

### Cell culture and reagents

HepG2, Huh7, Huh7.5 and Huh7.5M cells were cultured in DMEM containing 10% fetal calf serum, with or without 1× MEM nonessential amino acids and appropriate antibiotics. Transfections were performed using either X-fect reagent (Clontech) or Lipofectamine 2000 (Thermo Fisher), according to manufacturer’s protocols. Geneticin was from Thermo Fisher. TGF-β, U0126 and p38MAPK VIII inhibitors were from Merck Millipore. All antibodies except β-tubulin were from Cell Signaling Technologies, while β-tubulin antibody was from Thermo Fisher.

### Plasmids, over-expression and knock-down

Coding sequence of Snail, Slug, Zeb1 and Twist were amplified from MGC cDNA clones (Thermo Fisher) and cloned into pcDNA 4/TO vector. E-Cadherin and Vimentin coding sequences were amplified from Huh7.5 or Huh7.5M cDNAs and cloned into pcDNA 4/TO. All the clones were confirmed by sequencing. PL-SIN-Nanog-EGFP was from Addgene^49^(plasmid 21321). For ectopic over-expression, cells were transfected with pcDNA4/TO empty vectors or those expressing the desired protein. 48 hrs post transfection, expression of EMT markers was analyzed by immunobloting. Cells stably expressing any of these proteins were generated by selection under Zeocin. For Snail depletion, the cells were transfected with specific or non-targeting (NT) siRNA pool (Dharmacon). For Slug depletion, Huh7.5M cells were transfected with NT shRNA vector pools or those targeting Slug (Thermo Fisher). In both cases, cells were lysed at 72 hrs post transfection and processed for immunobloting.

### Proliferation Assay

Proliferation was measured by classical trypan blue exclusion method. Equal numbers of cells were seeded in dishes in duplicates and grown. At specific time intervals, cells were trypsinized, resuspended in media, mixed with equal volume of 0.4% trypan blue, and then were counted in a hemocytometer. Each sample was cultured in triplicate and counted separately. Average cell counts were plotted in graphs to study proliferation rate. Each graph represents data from three independent experiments. Cytotoxicity of inhibitors was measured by MTT assay. Average results from three independent experiments were represented graphically.

### Wound Heal Assay

Cells were grown in 35 mm dishes to 70% confluency. Scratches were made along the centre of the dish with tips. Cells were subsequently incubated in CO_2_ chamber and images were captured at regular intervals. Images were analyzed by Image J software. Distance between two approaching growth fronts at any time was measured and its ratio with that at 0 hour was represented as the percentage of cell migration.

### Real Time quantitative RT-PCR

Cells cultured normally or with inhibitors were harvested and total RNA was prepared by Trizol (Thermo Fisher). Complementary DNA strands were generated by reverse transcription using Primescript reverse transcriptase (Takara). 100 ng of cDNA samples were amplified with gene specific primers using SYBR Premix Ex Taq (Takara). Reactions were performed in Lightcycler 480 (Roche). Fold changes between the samples were calculated using normalized Cp values generated by the software. Average fold changes from a minimum of three independent experiments were graphically represented. Oligos used in the reactions are: Snail-For 5′-TCC GAC CCC AAT CGG AAG C-3′, -Rev 5′-CGG AGG TGG GAT GGC TGC-3′, SLUG-For 5′-TTC AAC GCC TCC AAA AAG CCA AAC TAC-3′, -Rev 5′-TAT GCT CCT GAG CTG AGG ATC TCT G-3′, Zeb1-For 5′-TAA GCG CAGAAA GCA GGC GAA-3′, -Rev 5′-TCT GTA ACA CTT TCT TCT TCC ACA ATA TGC-3′, CD44-For 5′-CGC AGC CTG GGG ACT CTG-3′, -Rev 5′-CGA GAG ATG CTG TAG CGA CCA-3′, CD24-For 5′-CAA TGG TGG CCA GGC TCG-3′, -Rev 5′-GCC AAC CCA GAG TTG GAA GTA CTC-3′, E-Cad-For 5′-GGA GCC GCA GCC TCT CGG CG-3′, -Rev 5′-CCC AGG ACG CGG CCT CTC TCC AG-3′, Vimentin-For 5′-GTG GAG CGC GAC AAC CTG-3′, -Rev 5′-GAC GTG CCA GAG ACG CAT TG-3′, GAPDH-For 5′-ATG GGG AAG GTG AAG GTC G-3′, -Rev 5′-GGG GTC ATT GAT GGC AAC AAT A-3′.

### Immunobloting and Confocal Immunofluorescence

Immunobloting was performed as described elsewhere^50^. Cells grown to 70% confluency were trypsinized, lysed in NP-40 lysis buffer and protein samples were quantified by BCA reagent (G Biosciences). For tumor tissue lysis, they were homogenized by tissue homogenizer followed by incubation with NP-40 lysis buffer. This was followed by centrifugation at 13000 rpm for 15 minutes and the supernatants were used as lysates for analyses. Equal quantities of samples were electrophoresed in SDS-PAGE and immunobloted. Detection was by Supersignal West Pico and Femto reagents (Thermo Fisher). β-tubulin immunoblots served as normalization control.

For confocal immunofluorescence, cells cultured in chamber slides were washed with 1× PBS, fixed in 4% paraformaldehyde and permeabilized with 0.5% Triton-X100. The slides were then blocked with 2% BSA and later incubated overnight with primary antibodies at 4 °C. Following this, the slides were washed and incubated with secondary antibodies conjugated with Alexa fluor 488 (Jackson Immuno Research Laboratories Inc.) for 1 hr at RT, washed and mounted with Vectashield mounting medium containing propidium iodide (Vector Laboratories Inc.) for nuclei staining. Fluorescence was analyzed using confocal laser-scanning microscope (Leica TCS SP5) with 40× oil immersion objective and 543-nm HeNe and 488-nm argon lasers. Images were captured and analyzed using LAS AF software.

### Anchorage Independence Growth (AIG) Assay

AIG assay was performed on poly(HEMA) coated 96 well plates. Wells were pre-coated with 30 mg/ml poly(HEMA) in absolute ethanol and allowed to dry for 48 hrs. 8 × 10^3^ cells were seeded into the coated wells and were allowed to grow for 96 hrs. At 96 hrs post seeding, growth of cells was measured by MTT reduction assay following established protocol^51^. In studies using inhibitors, cells suspended in media containing the respective inhibitors were grown in poly(HEMA) coated plates and at 72 hours post-seeding, MTT assay was performed. Spheroids formed were observed under phase contrast microscope.

### Subcellular fractionation

Cells were grown to 70% confluency, washed twice with ice-cold 1× PBS and incubated with ice-cold cytosol fraction buffer (150 mM NaCl, 50 mM Tris (pH-8.0), 0.7% NP-40, Protease inhibitor cocktail) on ice for 5 min. The soluble fraction was centrifuged at 3500 rpm for 10 min at 4 °C after which the supernatant served as cytosolic fraction. Cells were washed with ice-cold wash buffer (Cytosolic fraction buffer without 0.7% NP-40). The insoluble fraction was lysed with NP-40 lysis buffer and served as nuclear fraction. Purity of fractions was checked by immunobloting β-tubulin for cytosolic fraction and Lamin A/C for nuclear fraction.

### Mouse Tumor Xenograft Assay

3 × 10^6^ cells resuspended in equal volume of reduced serum matrigel (BD Biosciences) were injected into the right flank area of nude mice subcutaneously. Tumor volumes were measured using an electronic vernier caliper. Volumes were calculated according to the formula ½ Length × Width^2^. Six independent experiments were performed comprising twenty-eight animals. All the experiments had approval from the CCMB Animal Ethics Committee (50 /2014). The experiments were performed in accordance with the approved protocols and the animal received good human care.

For culturing tumor-derived cells, tumors were surgically removed after sacrificing the mice. Tumors were minced thoroughly after washing with sterile 1× PBS and were incubated with 0.25% trypsin-EDTA for 30 min at 37 °C. Subsequently, the minced pieces were filtered through cell strainer (BD Biosciences, San Diego, CA, USA) with 100 micron porosity. Filtrate was centrifuged at 1000 rpm for 2 min and the pellet that contained cells was resuspended in growth media and cultured as described earlier. For histopathology, tumor tissue as well as organs were fixed with formalin (10% v/v in PBS), dehydrated and paraffin embedded. Sections (4 μm) were stained with Hematoxylin–Eosin (HE) or Masson’s Trichrome^52^ Staining according to standard histological procedures.

### Statistical analyses

In all experiments, expect *in vivo* studies, statistical analyses were performed using Student’s t-test. Error bars were represented as Standard error ± mean (SEM). *In vivo* data was analyzed using Graphpad prism 5 software and statistical significance was calculated by unpaired Student’s t-test.

## Additional Information

**How to cite this article**: Vedagiri, D. *et al*. An Atypical System for Studying Epithelial-Mesenchymal Transition in Hepatocellular Carcinoma. *Sci. Rep.* **6**, 26282; doi: 10.1038/srep26282 (2016).

## Supplementary Material

Supplementary Information
