# Supplementary material for: An Atypical System for Studying Epithelial-Mesenchymal Transition in Hepatocellular Carcinoma
Source: Sci Rep. 2016 May 20;6:26282. doi: 10.1038/srep26282 (PMC4873837; doi:10.1038/srep26282)
Supplement: Supplementary Information [file srep26282-s1.pdf]

An Atypical System for Studying Epithelial-Mesenchymal Transition in Hepatocellular Carcinoma

Dhiviya Vedagiri[1]†, Hiren Vasantrai Lashkari[1]†, Abubakar Siddiq Mangani[1], Jerald Mahesh Kumar[1], Jedy Jose[1], Avinash Raj Thatipalli[1] and Krishnan Harinivas Harshan[1]*

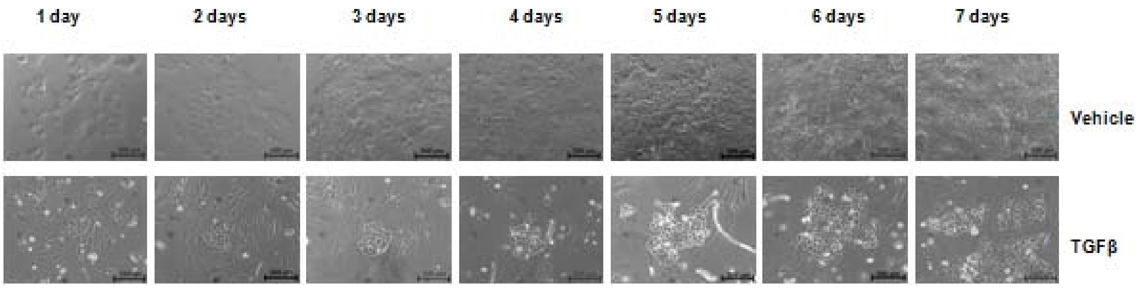

Supplementary Fig. S1. Microscopic analysis of Huh7.5 cultures treated with TGFβ for different time intervals. Cells seeded in six-well plates were treated with 2ng/ml of TGFβ or with vehicle at 24 hrs post seeding. Images were captured at every subsequent 24 hrs.

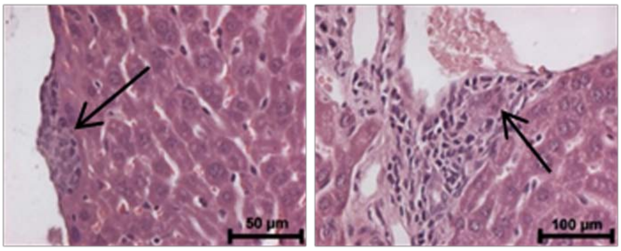

Supplementary Fig. S2. Histopathological analysis of liver tissues from mice injected with Huh7.5M cells. Tissue sections were stained with HE and imaged under microscope. Scale bars are shown in the images.

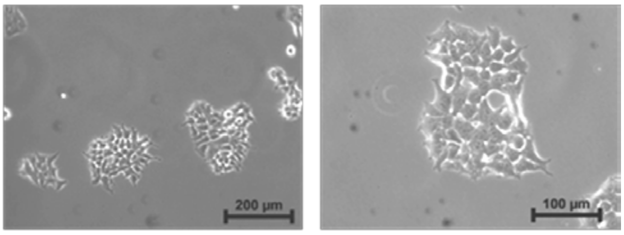

Supplementary Fig. S3. Reacquisition of mesenchymal morphology of tumor derived Huh7.5M cells. Cells generated from tumor tissues were seeded in cell culture dishes and imaged under phase contrast microscope.

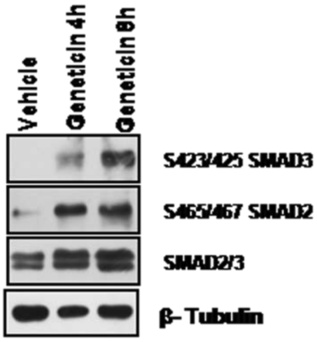

Supplementary Fig. S4. Regulation of canonical TGFβ signalling in Huh7.5 cells by geneticin analyzed by immunobloting. Cultured cells were incubated with geneticin for specific time intervals as described in the figure.

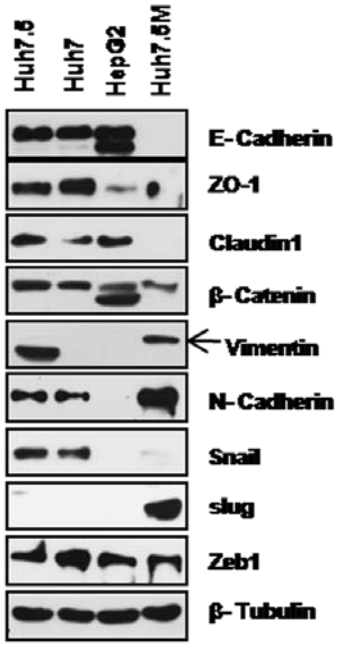

Supplementary Fig. S5. Comparison of EMT marker expression among Huh7.5, Huh7, HepG2 and Huh7.5M by immunobloting. All cells were cultured similarly and harvested for lysate preparation.

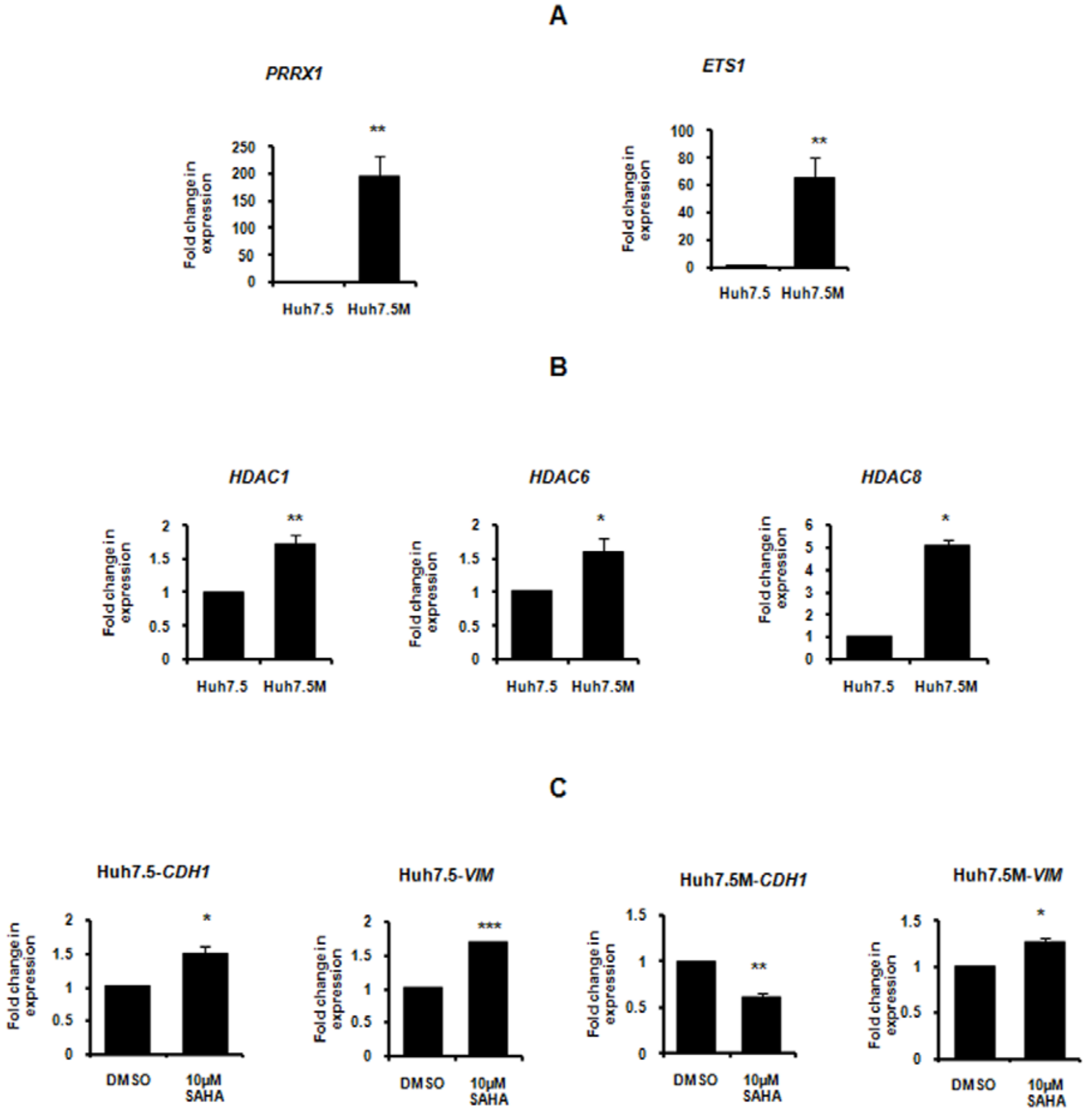

Supplementary Fig. S6. (A) Analysis of *Prrx1* and *Ets1* mRNA expression in Huh7.5 and Huh7.5M cells by qRT-PCR . RNA prepared from the respective cultured cells were converted to cDNA which was subjected to qPCR with specific primers. (B) HDAC1, 6 and 8 expression levels analyzed in Huh7.5 and Huh7.5M cells by qRT-PCR. (C) Analysis of expression of E-Cadherin and Vimentin mRNA expression upon HDAC inhibition by 10 µM SAHA, by qRT-PCR. Cells were treated with the inhibitor for 24 hrs before RNA preparation and qRT-PCR.
